# Sensitivity to thyroid hormone and risk of components of metabolic syndrome in a Chinese euthyroid population

**DOI:** 10.1111/1753-0407.13441

**Published:** 2023-07-10

**Authors:** Fang Lv, Xiaoling Cai, Yufeng Li, Xiuying Zhang, Xianghai Zhou, Xueyao Han, Linong Ji

**Affiliations:** ^1^ Department of Endocrinology and Metabolism Peking University People's Hospital Beijing China; ^2^ Department of Endocrinology Beijing Pinggu Hospital Beijing China

**Keywords:** adipose tissue, metabolic syndrome, sensitivity to thyroid hormone, skeletal muscle, 甲状腺激素敏感性, 代谢综合征, 骨骼肌, 脂肪组织

## Abstract

**Introduction:**

To evaluate the association of sensitivity to thyroid hormone with metabolic syndrome (MetS) and its components in a Chinese euthyroid population.

**Methods:**

A total of 3573 participants from Pinggu Metabolic Disease Study were analyzed. Serum‐free triiodothyronine (FT3), free thyroxine (FT4), thyrotropin (TSH), total adipose tissue (TAT), visceral adipose tissue (VAT), subcutaneous adipose tissue (SAT) area of abdominal, and lumbar skeletal muscle area (SMA) were measured. Central thyroid hormone resistance was calculated by the Thyroid Feedback Quantile‐based Index (TFQI) and Chinese‐referenced Parametric TFQI (PTFQI), Thyrotroph T4 Resistance Index (TT4RI) and TSH Index (TSHI). Peripheral thyroid hormone resistance was assessed by FT3/FT4 ratio.

**Results:**

Higher values of TSHI (odds ratio [OR] = 1.167, 95% confidence interval [CI]: 1.079–1.262, *p* < .001), TT4RI (OR = 1.115, 95% CI: 1.031–1.206, *p* = .006), TFQI (OR = 1.196, 95% CI: 1.106–1.294, *p* < .001), PTFQI (OR = 1.194, 95% CI: 1.104–1.292, *p* < .001), and lower values of FT3/FT4 ratio (OR = 0.914, 95% CI: 0.845–0.990, *p* = .026) were associated with MetS. Increased levels of TFQI and PTFQI were associated with abdominal obesity, hypertriglyceridemia, and hypertension. Increased levels of TSHI and TT4RI were associated with hypertriglyceridemia, abdominal obesity, low high‐density lipoprotein cholesterol. Reduced levels of FT3/FT4 ratio were associated with hyperglycemia, hypertension, and hypertriglyceridemia. The levels of TSHI, TFQI, and PTFQI were negatively related to SMA and positively related to VAT, SAT, and TAT (all *p* < .05).

**Conclusions:**

Reduced thyroid hormone sensitivity was associated with MetS and its components. Impaired thyroid hormone sensitivity might affect the distribution of adipose tissue and muscle.

## Graphical abstract

**Highlights**

- Mild acquired impaired thyroid hormone sensitivity was widespread among the general populations.
- Reduced thyroid hormone sensitivity was related with metabolic syndrome and its components.
- Impaired thyroid hormone sensitivity might affect the distribution of adipose tissue and muscle.

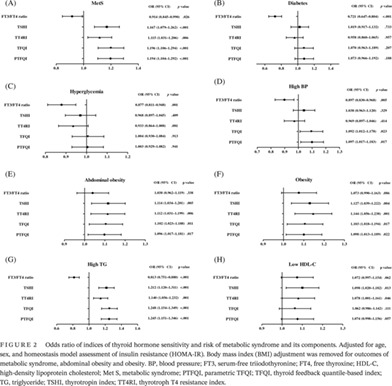

## 摘要

目的: 评估甲状腺功能正常人群中甲状腺激素敏感性与中国代谢综合征(MetS)及其组分的关系。

方法: 对平谷代谢性疾病研究中的3573名参与者进行分析。测量血清游离三碘甲状腺原氨酸(FT3)、游离甲状腺素(FT4)和促甲状腺激素(TSH)，计算腹部及腰部骨骼肌面积、内脏脂肪组织(VAT)、皮下脂肪组织(SAT)和全身脂肪面积(TAT)，通过甲状腺反馈量化的甲状腺激素抵抗指数(TFQI)、基于中国参考参数的参数TFQI(PTFQI)、促甲状腺素T4抵抗指数(TT4RI)以及TSH指数(TSHI)，计算周围甲状腺激素抵抗，即FT3/FT4比值。

结果: 较高的TSHI(OR=1.167，95%CI: 1.079，1.262，p<0.001)、TT4RI(OR=1.115，95%CI: 1.031，1.206，p=0.006)、TFQI(OR=1.196，95%CI: 1.106，1.294，p<0.001)、PTFQI(OR=1.194，95%CI: 1.104，1.292，p<0.001)以及较低的FT3/FT4比值(OR=0.914，95%CI: 0.845，0.990，p=0.026)与MetS相关。TFQI和PTFQI水平的升高与腹部肥胖、高三酰甘油血症和高血压相关。TSHI和TT4RI水平的升高与高三酰甘油血症、腹部肥胖、低高密度脂蛋白胆固醇(HDL‐C)相关。FT3/FT4比值的降低与高血糖、高血压和高三酰甘油血症相关。TSHI、TFQI和PTFQI水平与SMA呈负相关，与VAT、SAT和TAT呈正相关(均p＜0.05)。

结论: 甲状腺激素敏感性的降低与MetS及其组分相关。甲状腺激素敏感性的降低可能影响脂肪组织的分布和肌肉的改变。

## 1 INTRODUCTION

Metabolic syndrome (MetS) is a common metabolic and endocrine disease consisting of a cluster of cardiovascular risk factors such as abdominal obesity, hyperglycemia, dyslipidemia, and hypertension.^1^, ^2^ MetS is a serious health problem, and the prevalence of MetS has increased dramatically among adults worldwide.^1^

In terms of the relationship between thyroid hormones (THs) and MetS, it was found that hyperthyroidism and hypothyroidism, both overt and subclinical, might be associated with the risk of MetS.^3^, ^4^ However, the relationship between the normal range of THs (serum‐free thyroxine [FT4], free triiodothyronine [FT3]) and MetS is inconsistent.^5^, ^6^, ^7^, ^8^ Recently, it was reported that co‐occurrence of high thyrotropin (TSH) and high THs, which represented a kind of mild acquired resistance to thyroid hormones (RTH), was widespread among the general population.^8^ Therefore, impaired sensitivity to thyroid hormone might provide an appropriate explanation for these conflicting results between TSH, FT4, and MetS. However, RTH could occur despite high FT3/FT4. For example, thyroid hormone cell membrane transport defect caused by monocarboxylate transporter 8 (MCT8) gene mutation would lead to elevated FT3 levels and reduced FT4 and reverse T3 (rT3).^9^

This study aimed to evaluate the association between TH sensitivity and MetS, along with its components, in a Chinese euthyroid population. We also tried to explore the mechanism between sensitivity to TH and MetS by analyzing insulin resistance and body composition.

## 2 METHODS

### 2.1 Study design

The present study was conducted using the data from the Pinggu Metabolic Disease Study. Ethical approval was obtained from Ethics Committee at Peking University People's Hospital. Written informed consent was obtained from all study populations before enrollment. This study included 4002 participants aged 26–76 years. The enrollment and procedure could be found in previous studies.^6^, ^10^ In the current study, we included patients with normal levels of TSH and FT4. Patients who had a history of thyroid surgery, took TH replacement therapy, and antithyroid drugs were excluded. Finally, 3573 euthyroid participants were included in our analysis.

### 2.2 Physical examination

Standardized questionnaires were obtained from all participants as described previously. Height and weight were measured using a height‐weight scale. Body mass index (BMI) was calculated as weight, in kilograms, divided by height, in m^2^. Waist circumference (WC) was measured with a flexible non‐elastic tape at the midpoint between the lower border of the rib cage and the iliac crest. Hip circumference (HC) was measured at the largest circumference of the buttocks. The waist‐to‐hip ratio was calculated as the ratio of WC to HC. Blood pressure (BP) was measured three times consecutively after at least 5 min rest in the sitting position. The mean value of the three measurements of BP was calculated.

### 2.3 Biochemical and hormone assays

Venous blood samples were obtained after at least 8 h of overnight fast. Alanine aminotransferase, aspartate aminotransferase, total cholesterol, low‐density lipoprotein cholesterol, high‐density lipoprotein cholesterol (HDL‐C), triglyceride (TG), fasting plasma glucose (FPG), and creatinine concentrations were measured by the automated biochemical instrument (Beckman Coulter UniCel DxC 800, Brea, CA, USA). Participants without known diabetes underwent an oral glucose tolerance test to evaluate the status of glucose tolerance. Participants with diabetes measured only FPG. Hemoglobin A1c (HbA1c) was tested by cation‐exchange high‐pressure liquid chromatography method (Adams A1c HA‐8160, Japan). Serum insulin was measured by a radioimmunoassay method (China Institute of Atomic Energy, Beijing, China). The homeostasis model assessment of insulin resistance (HOMA‐IR) was calculated as fasting insulin (mU/mL) × FPG (mmol/L)/22.5.

### 2.4 Assessment of thyroid function and indices of thyroid hormone sensitivity

THs, including TSH, FT4, FT3, and thyroid‐related antibodies were measured by using a supersensitive electrochemiluminescence immunoassay (Siemens Centaur XP, Germany). The reference ranges of the thyroid function were 11.45–23.17 pmol/L for FT4, 3.5–6.5 pmol/L for FT3, 0.55–4.78 μIU/mL for TSH, <60 IU/mL for thyroid peroxidase antibodies, and <60 IU/mL for thyroglobulin antibody. Euthyroidism was defined as serum FT4 and TSH concentrations within reference ranges, without history of thyroid surgery, taking antithyroid drugs, and receiving TH replacement therapy.

Indices of central sensitivity to TH, including TSH index (TSHI), Thyrotroph T4 Resistance Index (TT4RI), Thyroid Feedback Quantile‐based Index (TFQI), Parametric Thyroid Feedback Quantile‐based Index (PTFQI), and peripheral sensitivity to thyroid hormone (FT3/FT4 ratio) were calculated according to previous studies.^8^, ^11^, ^12^ TT4RI was calculated as FT4 (pmol/L) * TSH (mIU/L).^12^ was calculated as ln TSH (mIU/L) + 0.1345 * FT4 (pmol/L).^11^ For TT4RI and TSHI, the higher the values, the lower the central sensitivity to THs. TFQI was calculated as cumulative distribution function (cdf) fT4‐ (1 − cdfTSH). The cdf for a random variable at *x* gives the probability that the random variable *X* is less than or equal to that number *x*.^8^ The PTFQI was used to define an approximate TFQI adapted to a Chinese reference population. The PTFQI was calculated as Φ((FT4 − *μ*fT4)/*σ*FT4) − (1 − Φ((ln TSH − *μ*lnTSH)/*σ*ln TSH)), where *μ*FT4 = 16.3802, *σ*FT4 = 1.98049, *μ*ln TSH = 0.5865, and *σ*ln TSH = 0.43854 for the Chinese population. For TFQI and PTFQI, negative and positive values were regarded as increased and decreased sensitivity to FT4, respectively. (5) FT3/FT4 ratio was calculated as the ratio of FT3 to FT4. Higher FT3/FT4 indicated higher peripheral TH activity.

### 2.5 Body composition measurements

All population were examined with abdominal plain CT (GE 64‐slice CT scanner, USA). The quantification of skeletal muscle area (SMA) was measured at the level of the lumbar vertebrae 3 (L3) intervertebral disc space, which contained the psoas, paraspinal, and abdominal wall muscles. The skeletal muscle indexes (SMI) was calculated by normalizing the total skeletal muscle surface area by the height in m^2^. The quantification of abdominal fat was measured at the level of the L4‐5 intervertebral disc space. Visceral adipose tissue (VAT) and total adipose tissue (TAT) area were assessed by the Tissue Composition Module of the software (Mindways, Austin, TX, USA). Subcutaneous adipose tissue (SAT) was assessed as TAT–VAT.

### 2.6 Definition

Subjects were classified as underweight (BMI < 18.5 kg/m^2^), normal weight (18.5–23.9 kg/m^2^), overweight (24.0–27.9 kg/m^2^), and obese (≥28.0 kg/m^2^) according to BMI recommended by the Working Group on Obesity in China.^13^ MetS diagnosis was established using the Chinese Diabetes Society criteria.^14^

### 2.7 Statistical analysis

Clinical characteristics were expressed as mean ± SD for continuous variables or numbers and percentages for categorical variables. Chi‐square test or Fisher's exact test was conducted to assess differences in categorical variables between different groups. Shapiro–Wilk tests were conducted to verify the normal or skewed distributions of continuous variables. Student's *t* test, Mann–Whitney *U* test or Kruskal–Wallis *H* test were performed to compare continuous variables between different groups. Pearson or Spearman correlation coefficients were conducted to assess the relationships between indices of sensitivity to TH and body composition. Partial correlation analysis was conducted to correct the effects of possible influencing factors. The linear‐by‐linear association test was used to analyze the trend of metabolic parameters in different quantiles of indices of sensitivity to TH. The associations of different risk factors with the prevalence of MetS (or its components) were evaluated by using binary logistic regression analysis. The TFQI, PTFQI, TSHI, TT4RI, and FT3/FT4 ratio was assessed for a 1 − SD increase of each variable. We adjusted potential confounders of MetS and its components using three models. In model 1, age and sex were adjusted; in model 2, age, sex, and BMI were adjusted; in model 3, HOMA‐IR was further adjusted. The receiver operating characteristic curve (ROC) analysis was performed to assess the capability of indices of sensitivity to thyroid hormone to differentiate MetS from controls.

All statistical analysis were conducted by SPSS software version 25.0 for windows (SPSS Inc., Chicago, IL, USA). All analysis were two‐sided, and a *p* value <.05 was considered as significant.

## 3 RESULTS

### 3.1 General characteristics of participants

Our analysis included 3573 euthyroid participants with a mean age of 49.8 ± 11.7 years. The prevalence of MetS was 42.8% (48.3% in men and 37.1% in women). Compared to participants without MetS, participants with MetS had higher BMI, WC, and prevalence of hypertension, hyperglycemia, and dyslipidemia. The levels of TSH (2.06 ± 0.94 vs. 2.02 ± 0.94 μIU/mL), FT4 (16.49 ± 2.14 vs. 16.01 ± 2.08 pmol/L), TT4RI (33.62 ± 15.04 vs. 31.84 ± 14.58), TSHI (2.84 ± 0.49 vs. 2.74 ± 0.50), TFQI (0.01 ± 0.36 vs. −0.06 ± 0.36), and PTFQI (0.03 ± 0.38 vs. −0.06 ± 0.38) were significantly higher in participants with MetS compared with participants without MetS (all *p* < .05) (Data S1). The results were consistent in both men and women. Compared with participants without MetS, the levels of FT3 were higher in participants with MetS in men (5.17 ± 0.51 and 5.26 ± 0.53 pmol/L, *p* < .001) but not in women (4.78 ± 0.42 and 4.81 ± 0.48 pmol/L, *p* = 0.098);whereas the FT3/FT4 ratio was lower in participants with MetS in women (0.31 ± 0.04 and 0.30 ± 0.04, *p* = .002) but not in men (0.32 ± 0.05 and 0.32 ± 0.04, *p* = .975) (Table 1).

**TABLE 1:** Demographic and clinical characteristics of study population.

Variables	Total *N* = 3573	Men			Women		
		MetS *N* = 876	Non‐MetS *N* = 938	*p* value	MetS *N* = 652	Non‐MetS *N* = 1107	*p* value
Age (years)	49.8 ± 11.7	48.9 ± 11.4	50.5 ± 12.3	**.002**	54.9 ± 9.9	47.0 ± 11.5	**<.001**
BMI (kg/m^2^)	26.06 ± 3.80	28.13 ± 2.23	24.23 ± 3.11	**<.001**	28.49 ± 3.47	24.56 ± 3.35	**<.001**
WC (cm)	86.8 ± 10.8	95.6 ± 8.0	83.5 ± 58.5	**<.001**	92.2 ± 8.8	79.3 ± 8.9	**<.001**
HC (cm)	98.4 ± 7.2	101.8 ± 6.2	95.6 ± 6.3	**<.001**	101.7 ± 7.0	96.0 ± 6.5	**<.001**
WHR	0.88 ± 0.07	0.94 ± 0.05	0.87 ± 0.06	**<.001**	0.91 ± 0.07	0.83 ± 0.06	**<.001**
ALT (U/L)	24.0 ± 19.2	31.0 ± 17.5	24.0 ± 27.2	**<.001**	24.0 ± 15.8	18.4 ± 10.3	**<.001**
AST (U/L)	23.2 ± 11.7	24.8 ± 11.5	23.9 ± 15.5	**.002**	23.6 ± 11.7	20.9 ± 7.1	**<.001**
Cr (μmol/L)	61.6 ± 26.4	72.1 ± 27.5	70.0 ± 36.0	**<.001**	53.0 ± 10.1	51.1 ± 13.9	**<.001**
FPG (mmol/L)	6.06 ± 1.59	6.66 ± 1.91	5.81 ± 1.29	**<.001**	6.61 ± 1.92	5.46 ± 0.90	**<.001**
FINS (uIU/mL)	9.63 ± 6.39	12.59 ± 8.00	6.72 ± 3.92	**<.001**	12.67 ± 6.19	7.98 ± 4.82	**<.001**
HbA1c (%)	5.82 ± 0.92	6.07 ± 1.09	5.59 ± 0.77	**<.001**	6.25 ± 6.15	5.55 ± 0.51	**<.001**
HOMA‐IR	2.68 ± 2.28	3.78 ± 2.93	1.74 ± 1.13	**<.001**	3.77 ± 2.35	1.97 ± 1.66	**<.001**
SBP (mm Hg)	130 ± 18	135 ± 16	129 ± 17	**<.001**	137 ± 18	123 ± 17	**<.001**
DBP (mm Hg)	79 ± 11	84 ± 11	78 ± 11	**<.001**	81 ± 11	74 ± 10	**<.001**
Cholesterol (mmol/L)	4.91 ± 0.97	4.98 ± 1.00	4.79 ± 0.88	**<.001**	5.15 ± 1.10	4.81 ± 0.91	**<.001**
Triglyceride (mmol/L)	1.58 ± 1.38	2.53 ± 1.99	1.06 ± 0.69	**<.001**	2.07 ± 1.22	0.96 ± 0.54	**<.001**
LDL‐C (mmol/L)	2.87 ± 0.80	2.84 ± 0.82	2.84 ± 0.77	.559	2.99 ± 0.85	2.84 ± 0.78	**<.001**
HDL‐C (mmol/L)	1.16 ± 0.31	0.97 ± 0.22	1.24 ± 0.24	**<.001**	1.07 ± 0.22	1.30 ± 0.30	**<.001**
TSH (μIU/mL)	2.04 ± 0.94	1.90 ± 0.87	1.83 ± 0.87	**.049**	2.29 ± 0.99	2.18 ± 0.97	**.014**
FT4 (pmol/L)	16.22 ± 2.12	16.87 ± 2.16	16.58 ± 2.16	**.006**	15.99 ± 2.01	15.52 ± 1.87	**<.001**
FT3 (pmol/L)	5.00 ± 0.53	5.26 ± 0.53	5.17 ± 0.51	**<.001**	4.81 ± 0.48	4.78 ± 0.42	.098
TPOAb (+) (%)	11.1	8.2	8.2	1.000	14.3	14.1	.944
TgAb (+) (%)	10.1	4.1	3.9	.905	16.4	16.3	.947
FT3/FT4 ratio	0.31 ± 0.04	0.32 ± 0.04	0.32 ± 0.05	.975	0.30 ± 0.04	0.31 ± 0.04	**.002**
TSHI	2.78 ± 0.50	2.81 ± 0.49	2.73 ± 0.51	**<.001**	2.88 ± 0.50	2.76 ± 0.49	**<.001**
TT4RI	32.60 ± 14.80	31.66 ± 14.21	29.93 ± 13.89	**.006**	36.26 ± 15.71	33.46 ± 14.95	**<.001**
TFQI	−0.03 ± 0.37	0.01 ± 0.36	−0.05 ± 0.36	**<.001**	0.02 ± 0.37	−0.09 ± 0.36	**<.001**
PTFQI	−0.018 ± 0.384	0.029 ± 0.381	−0.036 ± 0.391	**<.001**	0.036 ± 0.382	−0.071 ± 0.372	**<.001**
Diabetes (%)	15.1	27.1	7.0	**<.001**	28.8	4.2	**<.001**
Hypertriglyceridemia (%)	29.4	62.3	8.1	**<.001**	55.1	6.1	**<.001**
Obesity (%)	28.4	48.2	10.4	**<.001**	52.0	14.2	**<.001**
Abdominal obesity (%)	47.6	82.6	18.3	**<.001**	84.7	22.9	**<.001**
Hypertension (%)	68.3	86.0	59.1	**<.001**	89.9	49.3	**<.001**

*Note*: Data are expressed as means ± SD for continuous data or as *n* (%) for categorical data. Bold indicates statistically significant value.

Abbreviations: ALT, aminotransferase; AST, aspartate aminotransferase; BMI, body mass index; Cr, creatinine; DBP, diastolic blood pressure; FINS, fasting insulin; FPG, fasting plasma glucose; HbA1c, hemoglobin A1c; HC, hip circumference; HDL‐C, high‐density lipoprotein cholesterol; HOMA‐IR: homeostasis model assessment of insulin resistance; LDL‐C, low‐density lipoprotein cholesterol; MetS, metabolic syndrome; PTFQI, parametric TFQI; SBP, systolic blood pressure; TFQI, thyroid feedback quantile‐based index; TgAb, thyroglobulin antibody; TPOAb, thyroid peroxidase antibodies; TSHI, thyrotropin index; TT4RI, thyrotroph T4 resistance index; WC, waist circumference; WHR, waist‐to‐hip ratio.

**FIGURE 1:**
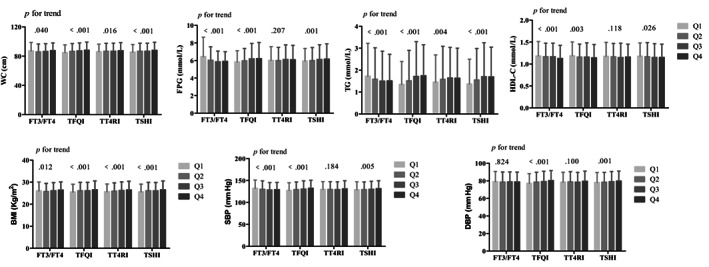
Metabolic parameters in different quantiles of indices of sensitivity to thyroid hormone. BMI, body mass index; DBP, diastolic blood pressure; FPG, fasting plasma glucose; FT3, serum‐free triiodothyronine; FT4, free thyroxine; HDL‐C, high‐density lipoprotein cholesterol; TFQI; TFQI, thyroid feedback quantile‐based index; TG, triglyceride; TSHI, thyrotropin index; TT4RI, thyrotroph T4 resistance index; SBP, systolic blood pressure; WC, waist circumference; WC, waist circumference.

The WC, FPG, TG, HDL‐C, BMI, and systolic BP were significantly reduced with the FT3/FT4 quartiles (all *p* for trend <.05). The WC, FPG, TG, BMI, and BP significantly increased, whereas HDL‐C decreased with the TFQI and TSHI quartiles (all *p* for trend <.05). The WC, TG, and BMI significantly increased with the TT4RI quartiles (all *p* for trend <.05) (Figure 1).

### 3.2 Association between sensitivity to thyroid hormone indices and the risk of MetS

**FIGURE 2:**
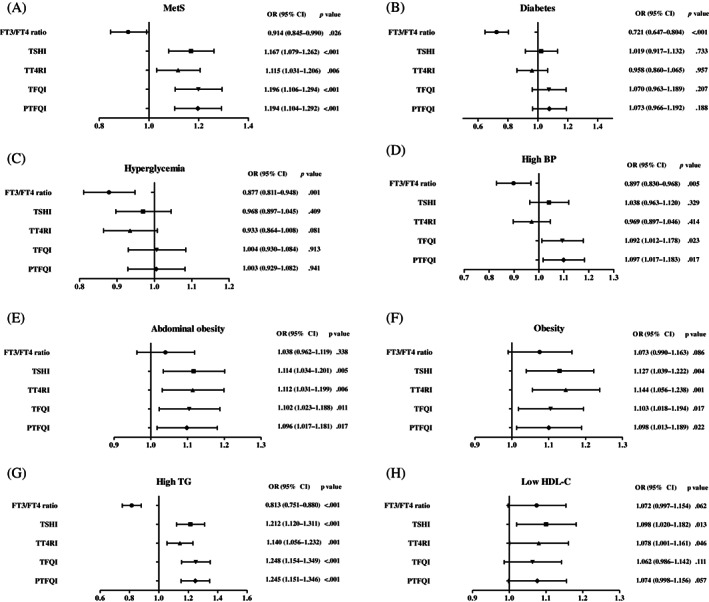
Odds ratio of indices of thyroid hormone sensitivity and risk of metabolic syndrome and its components. Model 1, adjusted for age and sex; model 2, adjusted for age, sex and BMI; model 3, adjusted for age, sex, BMI and HOMA‐IR; model 4, adjusted for age, sex, and HOMA‐IR. BMI adjustment was removed for outcomes of metabolic syndrome and high WC. Odds ratio of indices of thyroid hormone sensitivity and (A) metabolic syndrome. (B) diabetes. (C) hyperglycemia. (D) hypertension. (E) abdominal obesity. (F) obesity. (G) high TG. (H) low HDL‐C.

The odds ratio (OR) and 95% confidence interval [CI] of MetS and its components for each 1 SD increase in FT3/FT4 ratio, TT4RI, TSHI, TFQI, and PTFQI in the euthyroid population was presented in Figure 2 and Table 2. Higher levels of TSHI (OR = 1.167, 95% CI: 1.079–1.262, *p* < .001), TT4RI (OR = 1.115, 95% CI: 1.031–1.206, *p* = .006), TFQI (OR = 1.196, 95% CI: 1.106–1.294, *p* < .001) and PTFQI (OR = 1.194, 95% CI: 1.104–1.292, *p* < .001) were significantly associated with MetS even after adjusting for age, gender, and HOMA‐IR. No association was found between the FT3/FT4 ratio and risk of MetS after adjusting for age and sex (OR = 0.956, 95% CI: 0.893–1.023, *p* = .195), but the association was significant after further adjusting for HOMA‐IR (OR = 0.914, 95% CI: 0.845–0.990, *p* = .026).

**TABLE 2:** Odds ratio of indices of thyroid hormone sensitivity and risk of metabolic syndrome and its components.

	FT3/FT4 (+1SD)		TSHI (+1SD)		TT4RI (+1SD)		TFQI (+1SD)		PTFQI (+1SD)	
	OR, 95% CI	*p* value	OR, 95% CI	*p* value	OR, 95% CI	*p* value	OR, 95% CI	*p* value	OR, 95% CI	*p* value
Metabolic syndrome										
Model 1	0.956 (0.893–1.023)	.195	1.223 (1.142–1.309)	**<.001**	1.158 (1.082–1.239)	**<.001**	1.245 (1.163–1.333)	**<.001**	1.249 (1.167–1.337)	**<.001**
Model 4	0.914 (0.845–0.990)	**.026**	1.167 (1.079–1.262)	**<.001**	1.115 (1.031–1.206)	**.006**	1.196 (1.106–1.294)	**<.001**	1.194 (1.104–1.292)	**<.001**
Diabetes										
Model 1	0.751 (0.681–0.827)	**<.001**	1.105 (1.007–1.212)	**.034**	1.037 (0.945–1.136)	.445	1.149 (1.047–1.260)	**.003**	1.154 (1.051–1.266)	**.003**
Model 2	0.730 (0.661–0.806)	**<.001**	1.057 (0.962–1.163)	.248	0.993 (0.903–1.092)	.884	1.103 (1.003–1.212)	**.043**	1.108 (1.007–1.218)	**.035**
Model 3	0.721 (0.647–0.804)	**<.001**	1.019 (0.917–1.132)	.733	0.958 (0.860–1.065)	.957	1.070 (0.963–1.189)	.207	1.073 (0.966–1.192)	.188
Hyperglycemia										
Model 1	0.913 (0.852–0.979)	**.010**	1.053 (0.983–1.128)	.139	1.005 (0.938–1.076)	.894	1.085 (1.013–1.162)	**.020**	1.087 (1.015–1.165)	**.017**
Model 2	0.887 (0.826–0.953)	**.001**	1.005 (0.936–1.079)	.892	0.961 (0.894–1.032)	.274	1.040 (0.969–1.116)	.280	1.042 (0.970–1.118)	.260
Model 3	0.877 (0.811–0.948)	**.001**	0.968 (0.897–1.045)	.409	0.933 (0.864–1.008)	.081	1.004 (0.930–1.084)	.913	1.003 (0.929–1.082)	.941
Hypertension										
Model 1	0.918 (0.852–0.989)	**.025**	1.078 (1.001–1.161)	**.048**	1.002 (0.930–1.080)	.952	1.129 (1.048–1.216)	**.001**	1.135 (1.053–1.222)	**.001**
Model 2	0.896 (0.830–0.967)	**.005**	1.042 (0.966–1.124)	.283	0.971 (0.900–1.048)	.446	1.096 (1.016–1.183)	**.018**	1.101 (1.021–1.188)	**.012**
Model 3	0.897 (0.830–0.968)	**.005**	1.038 (0.963–1.120)	.329	0.969 (0.897–1.046)	.414	1.092 (1.012–1.178)	**.023**	1.097 (1.017–1.183)	**.017**
Abdominal obesity										
Model 1	1.051 (0.983–1.123)	.147	1.177 (1.101–1.258)	**<.001**	1.153 (1.079–1.233)	**<.001**	1.168 (1.093–1.248)	**<.001**	1.167 (1.092–1.247)	**<.001**
Model 4	1.038 (0.962–1.119)	.338	1.114 (1.034–1.201)	**.005**	1.112 (1.031–1.199)	**.006**	1.102 (1.023–1.188)	**.011**	1.096 (1.017–1.181)	**.017**
Obesity										
Model 1	1.060 (0.985–1.141)	.121	1.188 (1.104–1.279)	**<.001**	1.183 (1.101–1.272)	**<.001**	1.167 (1.085–1.255)	**<.001**	1.165 (1.083–1.254)	**<.001**
Model 4	1.073 (0.990–1.163)	.086	1.127 (1.039–1.222)	**.004**	1.144 (1.056–1.238)	**.001**	1.103 (1.018–1.194)	**.017**	1.098 (1.013–1.189)	**.022**
Hypertriglyceride**mia**										
Model 1	0.845 (0.785–0.911)	**<.001**	1.273 (1.182–1.370)	**<.001**	1.192 (1.109–1.281)	**<.001**	1.303 (1.211–1.403)	**<.001**	1.302 (1.210–1.402)	**<.001**
Model 2	0.809 (0.748–0.875)	**<.001**	1.220 (1.129–1.318)	**<.001**	1.145 (1.061–1.235)	**<.001**	1.257 (1.164–1.357)	**<.001**	1.255 (1.162–1.355)	**<.001**
Model 3	0.813 (0.751–0.880)	**<.001**	1.212 (1.120–1.311)	**<.001**	1.140 (1.056–1.232)	**.001**	1.248 (1.154–1.349)	**<.001**	1.245 (1.151–1.346)	**<.001**
Low HDL‐C										
Model 1	1.087 (1.014–1.166)	**.019**	1.156 (1.078–1.240)	**<.001**	1.128 (1.052–1.210)	**.001**	1.118 (1.043–1.199)	**.002**	1.131 (1.055–1.213)	**.001**
Model 2	1.064 (0.989–1.144)	.094	1.106 (1.028–1.190)	**.007**	1.083 (1.007–1.166)	**.033**	1.071 (0.996–1.152)	.064	1.084 (1.008–1.166)	**.030**
Model 3	1.072 (0.997–1.154)	.062	1.098 (1.020–1.182)	**.013**	1.078 (1.001–1.161)	**.046**	1.062 (0.986–1.142)	.111	1.074 (0.998–1.156)	.057

*Note*: Model 1, adjusted for age and sex; model 2, adjusted for age, sex, and BMI; model 3, adjusted for age, sex, BMI, and HOMA‐IR; model 4, adjusted for age, sex, and HOMA‐IR. BMI adjustment was removed for outcomes of metabolic syndrome and high WC. Bold indicates statistically significant value.

Abbreviations: BMI, body mass index; CI, confidence interval; FT3, serum‐free triiodothyronine; FT4, free thyroxine; HDL‐C, high‐density lipoprotein cholesterol; HOMA‐IR: homeostasis model assessment of insulin resistance; OR, odds ratio; PTFQI, parametric TFQI; TFQI, thyroid feedback quantile‐based index; TSHI, thyrotropin index; TT4RI, thyrotroph T4 resistance index; WC, waist circumference.

### 3.3 Association between sensitivity to thyroid hormone indices and the risk of components of MetS

After adjusting for age, sex, BMI, and HOMA‐IR, increased levels of TSHI and TT4RI were significantly correlated with hypertriglyceridemia, abdominal obesity, and low HDL‐C levels. Increased levels of TFQI and PTFQI were significantly associated with abdominal obesity, obesity, hypertension, and hypertriglyceridemia after adjusting for age, sex, BMI, and HOMA‐IR. The reduced levels of FT3/FT4 ratio was significantly associated with diabetes, hyperglycemia, and hypertriglyceridemia after adjusting for age, sex, BMI, and HOMA‐IR (Figure 2 and Table 2). However, all these indices had very low diagnostic sensitivity and specificity for MetS prediction and its components at the optimal cut‐points (Data S2).

### 3.4 Association between sensitivity to thyroid hormone and insulin resistance, indices of body composition

After adjustment of age, sex, and BMI, TT4RI was positively associated with SAT but not with VAT, TAT, VAT/TAT, SMA, and SMI. TSHI, TFQI, and PTFQI were negatively correlated with SMA and SMI and positively associated with VAT, SAT, and TAT. No significant correlations were found between TSHI and TFQI and VAT/TAT ratio. The FT3/FT4 ratio was negatively correlated with VAT, TAT, and VAT/TAT ratio. However, no significant correlations were found between FT3/FT4 ratio and SMA, SMI, and SAT. No significant association of sensitivity to TH with insulin resistance (all p > .05) (Data S3).

## 4 DISCUSSION

In this cross‐sectional study, we found reduced central TH sensitivity, including increased levels of TSHI, TT4RI, TFQI, and PTFQI, were correlated with the increased risk of MetS, abdominal obesity, and hypertriglyceridemia. We also found that the increased levels of TFQI and PTFQI were associated with the increased risk of hypertension, and the increased levels of TSHI and TT4RI were associated with low HDL‐C. Moreover, reduced peripheral sensitivity, indicated by a decreased FT3/FT4 ratio, was associated with an increased risk of MetS, hyperglycemia, hypertension, and hypertriglyceridemia.

RTH is a syndrome characterized by reduced sensitivity to TH. The majority of RTH cases are caused by mutations of TH receptor β, whereas a few RTH cases are caused by mutations of TH receptor α.^5^ In addition, defects of TH cell membrane transport (such as MCT8 defect),^15^ TH metabolism (such as selenocysteine insertion sequence binding protein 2 defect)^16^ are also causes of rare RTH.^4^ In 2019, Laclaustra et al put forward the hypothesis that a mild acquired resistance to THs was widespread among the general population, not a rare inherited defect.^8^

THs had effects on energy homeostasis, glucose metabolism, lipid, and BP.^17^, ^18^ Previous study reported that patients with RTHβ exhibited raised cholesterol and TG levels, reduced systemic insulin sensitivity and HDL‐C levels, and increased intramyocellular lipid and hepatic lipid.^19^ Thus, the reduced sensitivity to TH may be regarded as a newly developed risk factor for MetS and its components. The function of TH may explain the observed association between indices of resistance to TH and components of MetS. Participants with central RTH might have higher FT4 levels, thus reducing the risk of hyperglycemia and diabetes by improving glucose utilization and enhancing insulin sensitivity.^20^, ^21^ Besides the hypothalamus–pituitary–thyroid axis, the association between thyroid function and glucose metabolism could also be regulated by peripheral iodothyronine deiodinase (DIO) activity. In adipose tissue of obese subjects, the expression of DIO1 was upregulated through the stimulation of leptin.^22^ Various studies have suggested that THs may influence BP directly and indirectly. RTH would lead to reduction of the dilatation of the arterial smooth muscle, the nitric oxide availability, and endothelium‐dependent vasodilatation.^23^, ^24^, ^25^ In addition, TH had effects on fatty acid and cholesterol metabolism. TH could induce the expression of genes encoding proteins that played key roles in hepatic lipogenesis. TH could induce ketogenesis, and induce reverse cholesterol transport, by coupling autophagy to mitochondrial fat oxidation.^26^

In general population, the associations between TH sensitivity and metabolic parameters were reported inconsistently. Laclaustra et al reported the relationship between TFQI and the risk of obesity, MetS, diabetes, and diabetes‐related mortality in 5222 participants based on a sample representing the US population.^7^ In an Iranian population, TFQI was significantly associated with diabetes and high BP. TT4RI and TSHI were associated with high BP in euthyroid subjects.^25^ In a Chinese population‐based cross‐sectional study, reduced central sensitivity to THs (assessed by TT4RI, TSHI, TFQI, and PTFQI) was found to be associated with lower risk of prediabetes.^27^ Another cross‐sectional study in China suggested that FT3/FT4 ratio was negatively correlated with the risk of diabetes independent of confounding factors.^28^ In a retrospective study, central RTH was significantly correlated with higher glucose levels in patients with coronary heart disease.^29^ In our study, we observed that RTH was associated with high risk of MetS and some of its components, but different results were found for various TH sensitivity indices.

The mechanisms of RTH were complex and challenging. Likewise, the mild RTH in the general population could be variable in tissue expression and in severity. The “generalized,” “isolated pituitary,” and “peripheral tissue” resistance could represent tissue variability in the resistance to TH.^30^ Additionally, the variability in the severity of the resistance to TH could be explained by receptors position and difference in receptor sensitivity. Further studies on mechanisms underlying resistance to TH and MetS and its components were needed.

The underlying mechanism between TH sensitivity and metabolic parameters has not been fully elucidated. We tried to explore the association of sensitivity to TH, insulin resistance, and body composition. In this study, the TSHI and TFQI levels were negatively associated with skeletal muscle mass and positively associated with abdominal fat mass. FT3/FT4 was negatively associated with VAT and TAT. It was reported that the increase of adiposity, especially central adiposity, was one hallmark of MetS. Adiposity could cause an increase in cytokines and hormones and played important role in MetS via different pathways.^17^, ^22^ Impaired TH sensitivity might affect the distribution of adipose tissue and muscle, thus affecting the risk of MetS and its components.

However, the mechanism between TH sensitivity and indices of body composition remains a focus of debate. THs play crucial roles in the control of energy homeostasis and can influence body composition. TH induces energy utilization in white adipocytes through induction of uncoupling protein 1 expression.^31^ In contrast, the changes in body composition might influence TH levels. Weight loss resulting either from diet or from bariatric surgery induces a significant reduction in TSH and FT3.^32^ In addition, Nannipieri et al found reduced gene expression of TSH and FT3 receptors in both subcutaneous and visceral fat in obese subjects,^33^ which indicated the increase in plasma TSH and FT3 in obesity may be an attempt to cope with the peripheral resistance, as a consequence of the hypertrophy changes of adipocytes. Moreover, weight gain could lead to an increase in serum TSH levels and leptin, an adipose tissue‐derived hormone. Subsequently, leptin had an effect on thyrotropin‐releasing hormone or decreasing TH resistance by increasing the activity of type 1 deiodinase (D1) to result in the conversion of T4 to T3.^34^, ^35^ Thus, a variety of mechanisms may be involved in the associations of thyroid function with adiposity parameters. However, the pathogenesis of the relationship between sensitivity to TH and abdominal fat distribution has not yet been fully elucidated. Further longitudinal studies are needed to better understand the mechanisms by which TH sensitivity might affect abdominal fat distribution. Moreover, the altered expression of type 1 iodothyronine deiodinase, TH receptor, and TSH receptor in adipose tissue should be studied.

Our study had several strengths, such as the population‐based study design, the large sample size, evaluation of five different TH resistance indices, and corrected relevant confounding factors. However, our study has several limitations. First, the cross‐sectional design did not allow us to testify the cause and the effect relation between TH sensitivity indices and MetS and its components. Second, we did not account for all potential confounding factors that could influence the relationship between TH sensitivity and MetS, such as other medical conditions, medications, or lifestyle factors. Third, polymorphisms in the genes of RTH could modulate the expression of the mutated gene contributing to the clinical presentation of RTH. Incorporating genetic testing to identify the source of impaired TH sensitivity could potentially add value to our work.

## 5 CONCLUSION

In this study, we demonstrated the association between impaired central or peripheral RTH and the increased risk of MetS. We also found the relation between central or peripheral RTH and the increased risk of obesity, dyslipidemia, and hypertension. Our study provided evidence for the significance of RTH in their interactions with MetS in a Chinese population; the underlying mechanism needs to be explored further.

## AUTHOR CONTRIBUTIONS

Linong Ji and Xiaoling Cai conceptualized this study and designed the systematic review protocol; Fang Lv, Yufeng Li, Xiuying Zhang, Xueyao Han, and Xianghai Zhou performed the study selection and data extraction; Fang Lv performed the statistical analyses; Fang Lv and Xiaoling Cai prepared the outlines and wrote the manuscript. All authors contributed to the critical revision of manuscript drafts.

## FUNDING INFORMATION

This work was supported by National Natural Science Foundation of China (No. 81970698, No. 81900805, and No. 81970708), Beijing Natural Science Foundation (No. 7202216), and Peking University People's Hospital Research and Development Funds (Project RS2022‐03). The funding agencies had no roles in the study design, data collection or analysis, decision to publish or preparation of the manuscript.

## CONFLICT OF INTEREST STATEMENT

No potential conflicts of interest relevant to this article were reported.

## Supporting information


**DATA S1.** Indices of sensitivity to thyroid hormone in participants with and without MetS. Compared to participants without MetS, TSHI, TT4RI, TFQI, and PTFQI were significantly higher in participants with MetS (all *p* < .001). The FT3/FT4 ratio were similar in participants with and without MetS (*p* = .109). FT3, serum‐free triiodothyronine; FT4, free thyroxine; Met S, metabolic syndrome; PTFQI, parametric TFQI; TFQI, thyroid feedback quantile‐based index; TSHI, thyrotropin index; TT4RI, thyrotroph T4 resistance index.
**DATA S2.** ROC curves of indices of thyroid hormone sensitivity for metabolic syndrome and its components. (A) ROC curves of indices of thyroid hormone sensitivity for metabolic syndrome. (B) ROC curves of indices of thyroid hormone sensitivity for diabetes. (C) ROC curves of indices of thyroid hormone sensitivity for obesity. (D) ROC curves of indices of thyroid hormone sensitivity for abdominal obesity. (E) ROC curves of indices of thyroid hormone sensitivity for high BP. (F) ROC curves of indices of thyroid hormone sensitivity for high TG. (G) ROC curves of indices of thyroid hormone sensitivity for low HDL‐C. BP, blood pressure; HDL‐C, high‐density lipoprotein cholesterol; ROC, receiver operating characteristics; TG, triglyceride.
**DATA S3.** Association between sensitivity to thyroid hormone and insulin resistance, indices of body composition. After adjustment of age, sex, and body mass index.

## Data Availability

No additional data available.
