# Supplementary material for: Sensitivity to thyroid hormone and risk of components of metabolic syndrome in a Chinese euthyroid population
Source: J Diabetes. 2023 Jul 10;15(10):900–10. doi: 10.1111/1753-0407.13441 (PMC10590679; doi:10.1111/1753-0407.13441)
Supplement: Supplementary file 1 — DATA S1. Indices of sensitivity to thyroid hormone in participants with and without MetS. Compared to participants without MetS, TSHI, TT4RI, TFQI, and PTFQI were significantly higher in participants with MetS (all p < .001). The FT3/FT4 ratio were similar in participants with and without MetS (p = .109). FT3, serum‐free triiodothyronine; FT4, free thyroxine; Met S, metabolic syndrome; PTFQI, parametric TFQI; TFQI, thyroid feedback quantile‐based index; TSHI, thyrotropin index; TT4RI, thyrotroph T4 resistance index. DATA S2. ROC curves of indices of thyroid hormone sensitivity for metabolic syndrome and its components. (A) ROC curves of indices of thyroid hormone sensitivity for metabolic syndrome. (B) ROC curves of indices of thyroid hormone sensitivity for diabetes. (C) ROC curves of indices of thyroid hormone sensitivity for obesity. (D) ROC curves of indices of thyroid hormone sensitivity for abdominal obesity. (E) ROC curves of indices of thyroid hormone sensitivity for high BP. (F) ROC curves of indices of thyroid hormone sensitivity for high TG. (G) ROC curves of indices of thyroid hormone sensitivity for low HDL‐C. BP, blood pressure; HDL‐C, high‐density lipoprotein cholesterol; ROC, receiver operating characteristics; TG, triglyceride. DATA S3. Association between sensitivity to thyroid hormone and insulin resistance, indices of body composition. After adjustment of age, sex, and body mass index. [file JDB-15-900-s001.docx]

**Supplemental data 1. Indices of sensitivity to thyroid hormone in participants with and without MetS.**

Compared to participants without MetS, TSHI, TT4RI, TFQI and PTFQI were significantly higher in participants with MetS (all *p*<0.001). The FT3/FT4 ratio were similar in participants with and without MetS (*p*=0.109).

**Supplemental data 2. ROC curves of indices of thyroid hormone sensitivity for metabolic syndrome and its components.**

A. ROC curves of indices of thyroid hormone sensitivity for metabolic syndrome.

B. ROC curves of indices of thyroid hormone sensitivity for diabetes.

C. ROC curves of indices of thyroid hormone sensitivity for obesity.

D. ROC curves of indices of thyroid hormone sensitivity for abdominal obesity.

E. ROC curves of indices of thyroid hormone sensitivity for high BP.

F. ROC curves of indices of thyroid hormone sensitivity for high TG.

G. ROC curves of indices of thyroid hormone sensitivity for low HDL-C.


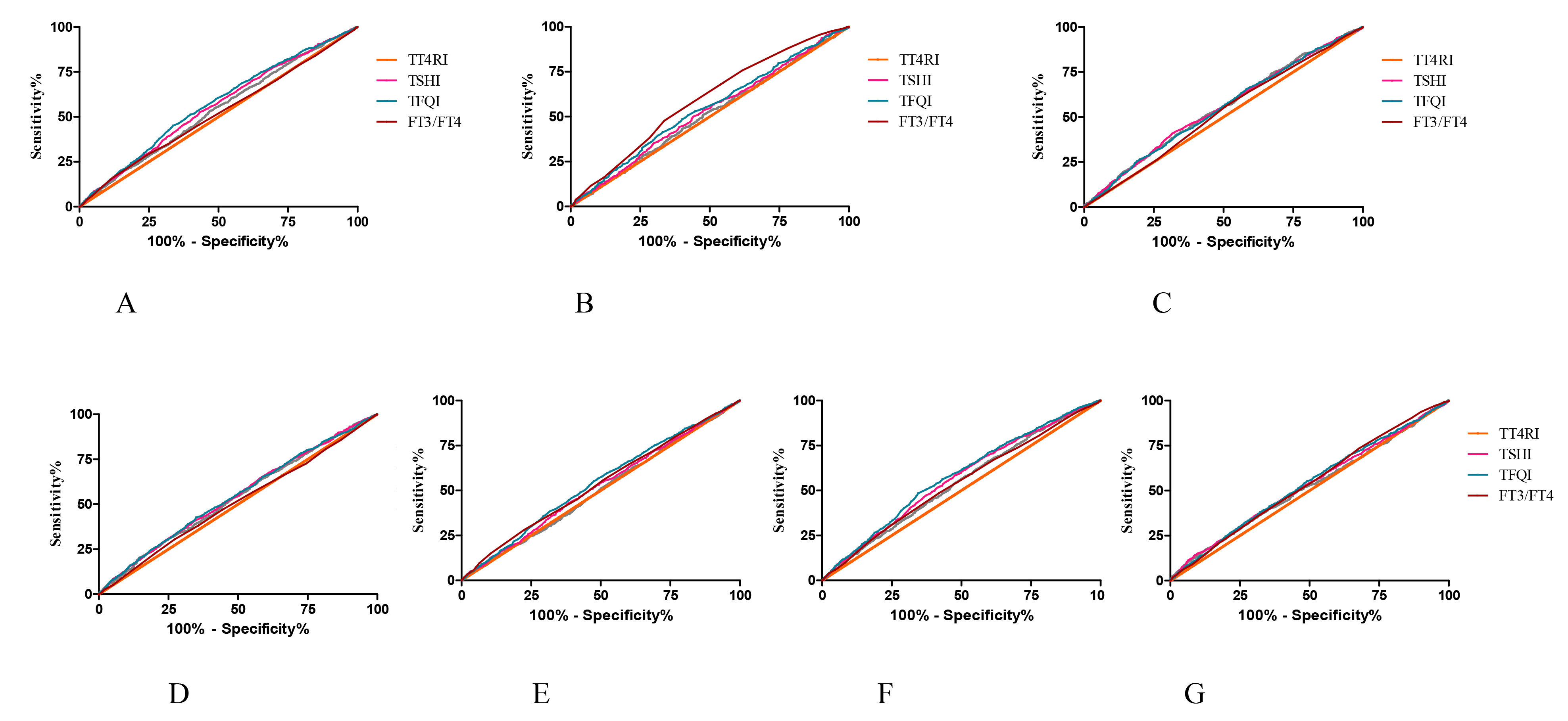


**Supplemental data 3. Association between sensitivity to thyroid hormone and insulin resistance, indices of body composition.**

|  | FT3/FT4 | | TSHI | | TT4RI | | TFQI | | PTFQI | |
| --- | --- | --- | --- | --- | --- | --- | --- | --- | --- | --- |
|  | r | *p* | r | *p* | r | *p* | r | *p* | r | *p* |
| TAT | -0.046 | **0.011** | 0.064 | **<0.001** | 0.032 | 0.079 | 0.075 | **<0.001** | 0.077 | **<0.001** |
| VAT | -0.068 | **<0.001** | 0.044 | **0.017** | 0.002 | 0.896 | 0.054 | **0.003** | 0.059 | **0.001** |
| SAT | 0.001 | 0.750 | 0.054 | **0.003** | 0.044 | **0.017** | 0.059 | **0.001** | 0.058 | **0.001** |
| VAT/TAT | -0.059 | **0.001** | <0.001 | 0.996 | -0.023 | 0.204 | 0.009 | 0.617 | 0.012 | 0.504 |
| SMA | 0.012 | 0.514 | -0.047 | **0.011** | -0.028 | 0.126 | -0.045 | **0.015** | -0.052 | **0.004** |
| SMI | 0.023 | 0.200 | -0.053 | **0.004** | -0.032 | 0.077 | -0.054 | **0.003** | -0.06 | **0.001** |
| HOMA-IR | -0.024 | 0.191 | 0.024 | 0.193 | 0.006 | 0.738 | 0.030 | 0.107 | 0.031 | 0.087 |

After adjustment of age, sex and BMI.
